# Orally delivered water soluble Coenzyme Q_10_ (Ubisol-Q_10_) blocks on-going neurodegeneration in rats exposed to paraquat: potential for therapeutic application in Parkinson’s disease

**DOI:** 10.1186/1471-2202-15-21

**Published:** 2014-01-31

**Authors:** Krithika Muthukumaran, Samantha Leahy, Kate Harrison, Marianna Sikorska, Jagdeep K Sandhu, Jerome Cohen, Corrine Keshan, Daniel Lopatin, Harvey Miller, Henryk Borowy-Borowski, Patricia Lanthier, Shelly Weinstock, Siyaram Pandey

**Affiliations:** 1 Chemistry & Biochemistry, University of Windsor, 401 Sunset Ave, Windsor, ON, Canada; 2 Psychology, University of Windsor, Windsor, ON, Canada; 3 Translational Bioscience, Human Health Therapeutics Portfolio, National Research Council Canada, Ottawa, ON, Ontario K1A 0R6, Canada; 4 Zymes LLC, Hasbrouck Heights, NJ, USA

**Keywords:** Parkinson’s disease, Oxidative stress, Neuronal cell death, Mitochondrial dysfunction, Water soluble CoQ_10_

## Abstract

**Background:**

Paraquat, still used as an herbicide in some parts of the world, is now regarded as a dangerous environmental neurotoxin and is linked to the development Parkinson’s disease (PD). Paraquat interacts with cellular redox systems and causes mitochondrial dysfunction and the formation of reactive oxygen species, which in turn, plays a crucial role in the pathophysiology of PD. Various antioxidant therapies have been explored with the expectations that they deliver health benefits to the PD patients, however, no such therapies were effective. Here we have tested the neuroprotective efficacy of a novel water-soluble CoQ_10_ (Ubisol-Q_10_), in a rat model of paraquat-induced neurodegeneration in order to evaluate its potential application in the management of PD.

**Results:**

We have developed a rat model of progressive nigrostriatal degeneration by giving rats five intraperitoneal injections of paraquat (10 mg/kg/injection), once every five days. Neuronal death occurred over a period of 8 weeks with close to 50% reduction in the number of tyrosine hydroxylase-positive cells. Ubisol-Q_10_, at 6 mg CoQ_10_/kg body weight/day, was delivered as a supplement in drinking water. The intervention begun after the completion of paraquat injections when the neurodegenerative process had already began and about 20% of TH-positive neurons were lost. Ubisol-Q_10_ treatment halted the progression of neurodegeneration and remaining neurons were protected. The outcomes were evaluated based on the number of surviving tyrosine hydroxylase-positive neurons in the substantia nigra region and improved motor skills in response to the Ubisol-Q_10_ intervention. To maintain this neuroprotection, however, continuous Ubisol- Q_10_ supplementation was required, if withdrawn, the neuronal death pathway resumed, suggesting that the presence of CoQ_10_ was essential for blocking the pathway.

**Conclusion:**

The CoQ_10_, given orally as Ubisol-Q_10_ in drinking solution, was effective in blocking the progression of neurodegeneration when administered therapeutically (post-toxin injection), at a much lower concentration than other previously tested oil soluble formulations and well within the acceptable daily intake of 12 mg/kg/day. Such unprecedented neuroprotection has never been reported before. These results are very encouraging and suggest that Ubisol-Q_10_ should be further tested and developed as a therapy for halting the progression of PD.

## Background

Parkinson’s disease (PD), the second most common neurodegenerative disorder, is characterised by the loss of dopaminergic (DA) neurons in the *substantia nigra pars compacta* (SNpc) region of the brain. PD affects approximately 1–2% of the population, above the age of 55 and with the steady growth of the ageing population, disease management is a growing concern for neurologists and other physicians. By the time the characteristic features of PD such as bradykinesia, rigidity, postural instability, and resting tremor become obvious, approximately 60-70% of DA neurons in the SNpc are lost [1]. Currently, there is no therapy available to halt the progression of this neurodegeneration. It has been possible, however, to alleviate the symptoms of the disease by providing dopamine replacement. Administration of levodopa is the most commonly utilized treatment for symptomatic relief [2], yet its prolonged application leads to drug induced dyskinesia, which severely affects the patient’s quality of life.

In the majority of cases the cause of PD remains unknown, but factors contributing to the pathogenesis of the disease are extensively studied. PD can be caused by environmental factors such as exposure to herbicides and pesticides or by genetic factors linked to gene mutations that increase the susceptibility to PD [3]. Although these genetic defects account for only 10% of PD cases, their identification brings about a better understanding of the disease pathophysiology and its progressive nature [4]. It is known that classical symptoms of PD can be caused by exposure to neurotoxin MPTP. In 1983 Langston’s group found PD like symptoms in young drug addicts who consumed heroin containing MPTP, a by-product in the synthesis of a synthetic heroin [5]. Later it was shown that MPTP injections cause selective loss of DA neurons in the SNpc region of certain strains of mice thereby creating animal models of PD [6-9]. Although MPTP is not an environmental toxin and humans are not commonly exposed to it, several epidemiological studies reveal a link between the use of herbicides and pesticides such as paraquat (PQ), maneb and rotenone and the incidence of PD [10]. It was subsequently discovered that the active metabolite of MPTP, MPP + and PQ have structural similarity. They enter the DA neurons via the dopamine transporter as well as trigger neurodegeneration [11]. Three independent studies in Texas, Taiwan and California show that exposure to PQ indeed causes an increased susceptibility to PD [12,13]. In rodents, PQ exposure leads to the loss of DA neurons in the SNpc region of the brain in a time and dose dependent manner [14]. Therefore, rat and mouse models of PQ-induced neurodegeneration have been developed to study the pathophysiology of the disease and to develop successful treatment strategies.

One consistent finding between the PD patients and animal models of PD (MPTP, PQ, rotenone) is the malfunctioning of complex I of the electron transport chain suggesting clearly, that mitochondrial dysfunction is at the centre of PD pathophysiology [4]. It seems that a blockade of complex I of the oxidative phosphorylation pathway by these toxins and the inability of DA neurons to cope with the excess of generated free radicals are the triggers of neuronal death. Therefore, it should be possible to interfere with the progression of neurodegenerative processes by applying antioxidants, such as CoQ_10_ and/or Vitamin E, which are capable of reducing the levels of free radicals. However, both these antioxidants are lipid soluble compounds, characterized by limited bioavailability and difficult to deliver systemically, especially to the brain. Numerous studies have shown that CoQ_10_ is effective in preventing cell death caused by toxins such as PQ, however, very high doses of CoQ_10_ (from oil soluble formulation available on the market) are required to provide neuroprotection *in vivo*[15]. Our collaborators at NRC (Ottawa, ON) have developed a nanomiscelle formulation of CoQ_10_ (Ubisol-Q_10_), which appears water soluble and contains CoQ_10_ and a derivatized form of α-tocopherol (vitamin E) [16,17]. The solubilization of CoQ_10_ is achieved due to amphipathic properties of PEG-derivatised α-tocopherol allowing the formation of stable and water soluble nanomicelles [16,17]. This formulation has been tested in several cell culture models and it has been shown to be efficient in protecting neurons from the toxic effects of PQ [18]. It has also been tested *in vivo* in rats exposed to PQ [14]. Prophylactic application of Ubisol-Q_10_ in this rat model of PD, provided as drinking solution prior to the PQ exposure and throughout the duration of experiments, clearly confirm its neuroprotective efficacy against PQ. The beneficial effects were achieved at a much lower dose of CoQ_10_ (6 mg/kg b.w.) compared to oil soluble formulation, which was used at 200 – 1600 mg/kg/day in mice [19].

Since PD is diagnosed when the symptoms appear and the neurodegeneration is already in progress, the prophylactic treatments, especially in sporadic cases of PD, are not relevent. Therefore, we designed a study to examine whether a therapeutic intervention with Ubisol-Q_10_ in rats already exposed to PQ could halt the on-going neurodegeneration and behavioural deterioration. Furthermore, we investigated whether a sustained supplementation of Ubisol-Q_10_ was needed to maintain the neuroprotection. Here we present our data showing that oral delivery of Ubisol-Q_10_, starting after the PQ injections, did halt neurodegeneration and prevented a loss of normal motor skills.

## Results

### Brain delivery of Coenzyme Q10

**Figure 1:**
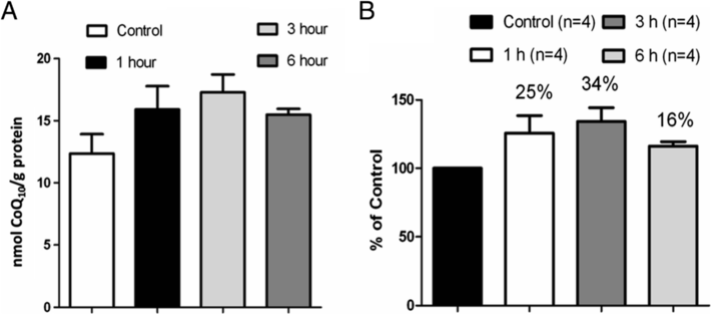
**Bioavailability of Ubisol – Q**_**10**_**. (A)** There is a gradual increase in the levels of CoQ_10_ in the rat brains that were sacrificed at the 1 and 3 hour time points following the 1 hour feeding with Ubisol – Q_10_ supplemented drinking water after the 24 hour water starvation in comparison with the control group that was fed with regular drinking water for 1 hour instead. The control group was sacrificed 1 hour following the water feeding. **(B)** There is a 25% increase in the level of CoQ_10_ at the 1 hour time point, 34% at the 3 hour time point and a decline to 16% at the 6 hour time point.

**Figure 2:**
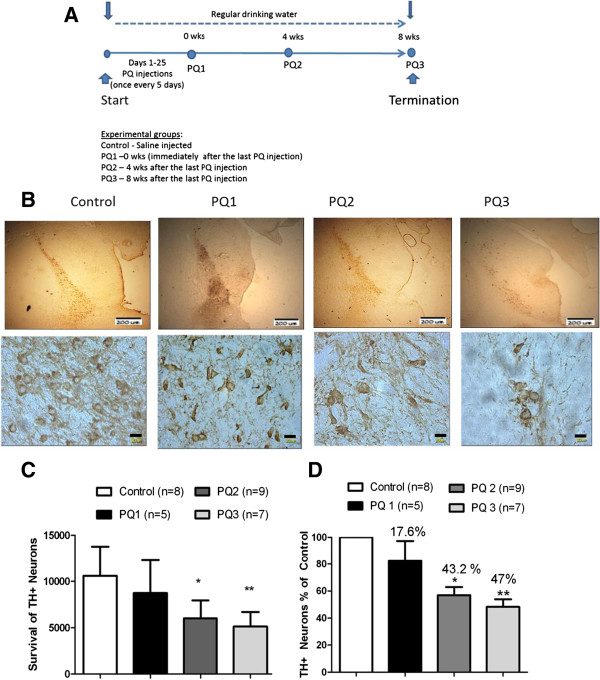
**Progressive loss of DA neurons following PQ injections. (A)** The experimental plan demonstrating the injection regime and treatment schedule. Immunohistochemistry performed using anti-tyrosine hydroxylase antibodies on the brain sections of animals. **(B)** Representative images of midbrain sections showing TH positive neurons at lower and higher magnifications from saline injected control group rats, PQ injected rats dissected 24 hours after the last injection (PQ1 group), PQ injected rats dissected four weeks after the last injection (PQ2 group), PQ injected rats dissected eight weeks after the last injection (PQ3 group). The area of SN which is to be counted is selected in every 6^th^ section of the midbrain (sectioned at 30 microns thickness). **(C)** The total number of TH positive neurons in the SNpc region was counted at higher magnification using the stereology software purchased from the Stereology Resource Centre, Inc., Florida. There is a significant decrease in the number of TH - positive neurons (*p < 0.05) in the rats sacrificed four weeks and (**p < 0.05) eight weeks after the last injection in comparison with saline injected control group and no significance in rats sacrificed immediately after the last injection verses saline injected control groups. **(D)** The percentage decrease in TH positive neurons between the saline injected control and PQ groups. There is 43.3% and 47% decrease in the TH positive neurons in the rats sacrificed four and eight weeks after the last injection and only a 17.8% decrease in the number of TH positive neurons in the rats sacrificed 24 hours after the last injection. The bars in the upper (low magnification) panel are 200 μm and in the lower (high magnification) panel 20 μm.

**Figure 3:**
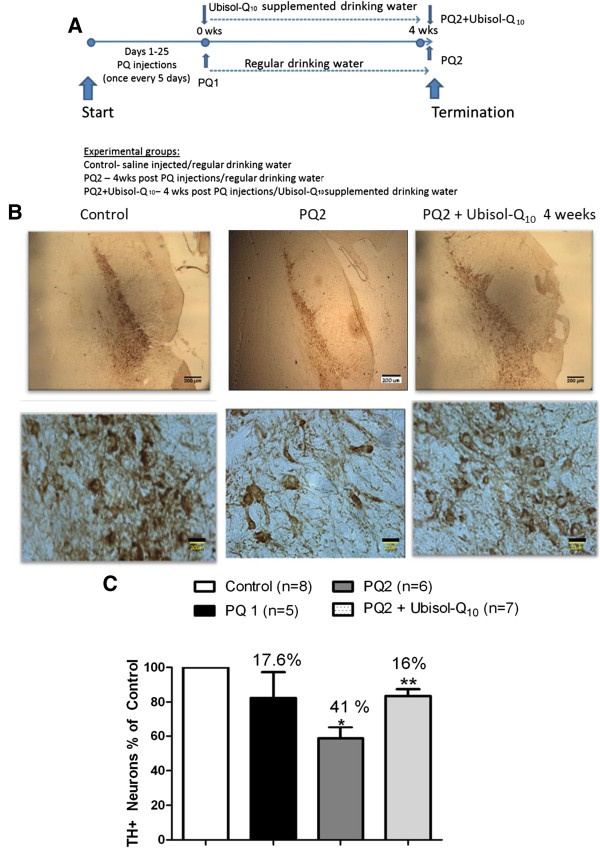
**Halting the progression of neurodegeneration by Ubisol – Q**_**10**_**. (A)** The experimental plan demonstrating the injection regime and treatment schedule. Immunohistochemistry performed using anti-tyrosine hydroxylase antibodies on the brain sections of animals. **(B)** Representative images of midbrain sections showing TH positive neurons at lower and higher magnifications from saline injected control group rats, PQ injected rats fed with regular drinking water dissected immediately after the last injection (PQ1), PQ injected rats fed with regular drinking dissected 4 weeks after last injection (PQ2), PQ injected rats fed with Ubisol – Q_10_ supplemented drinking water after last injection and dissected 4 weeks after last injection (PQ2 + Ubisol- Q_10_ 4 weeks). The area of SN which is to be counted is selected in every 6^th^ section of the midbrain (sectioned at 30 microns thickness). The total number of TH positive neurons in the SNpc region was counted at high magnification using the stereology software purchased from the Stereology Resource Centre, Inc., Florida. **(C)** The percentage decrease in TH positive neurons between the saline injected control group and the PQ injected treated and untreated groups. There is a significant 41% decrease in the TH positive neurons in the PQ2 group in comparison with the saline injected control group (*p < 0.05), whereas there is loss of 17% neurons in the Ubisol – Q_10_ treated group verses the control (**p < 0.05) indicating significant neuroprotection when compared to the PQ2 group. The bars in the upper (low magnification) panel are 200 μm and in the lower (high magnification) panel 20 μm.

**Figure 4:**
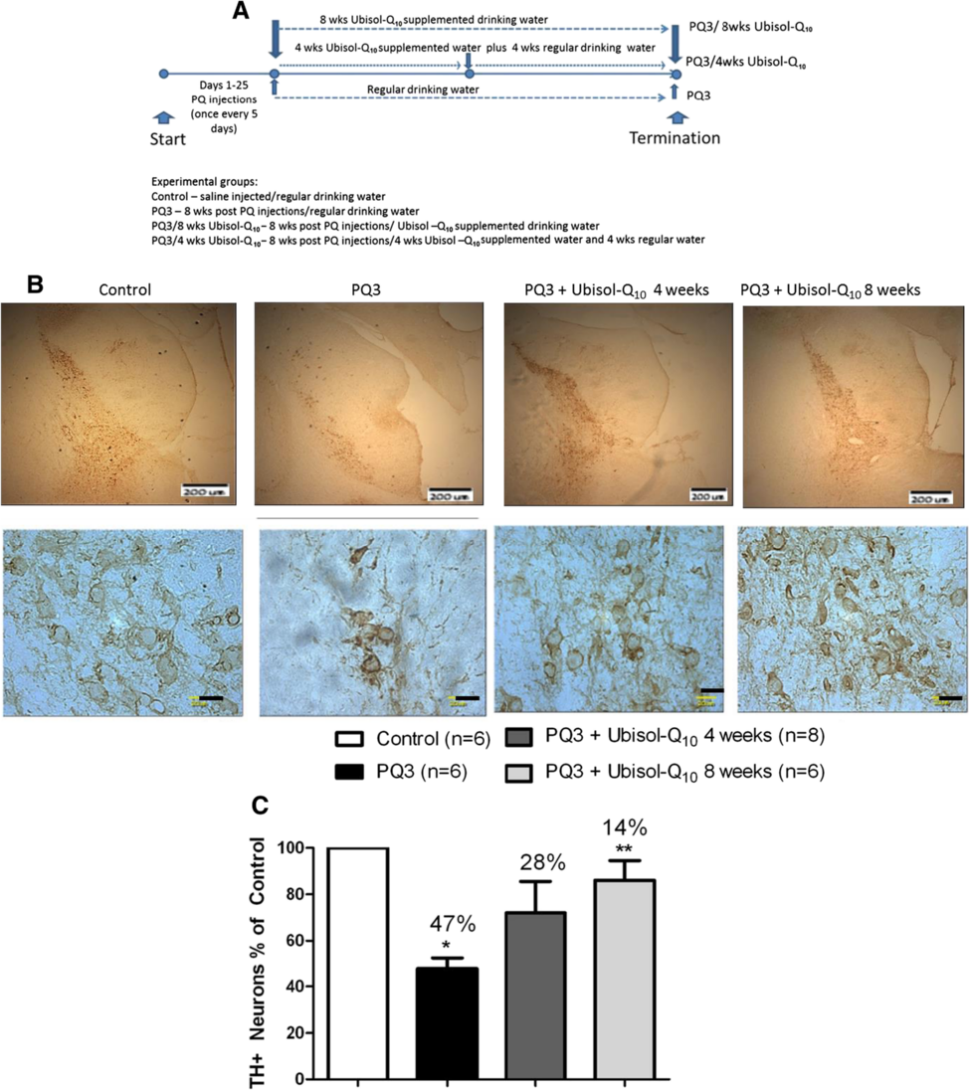
**Sustained feeding of Ubisol-Q**_**10 **_**is needed for neuroprotection. (A)** The experimental plan demonstrating the injection regime and treatment schedule. **(B)** Immunohistochemistry performed using anti-tyrosine hydroxylase antibodies on the brain sections of animals. Representative images of midbrain sections showing TH positive neurons at lower and higher magnifications from saline injected control group rats, PQ injected rats fed with regular drinking water dissected 8 weeks after last injection (PQ3), PQ injected rats fed with Ubisol – Q_10_ supplemented drinking water for 4 weeks followed by regular drinking water for 4 weeks after last injection with PQ and dissected 8 weeks after last injection (PQ3 + Ubisol – Q_10_ 4 weeks group), PQ injected rats fed with Ubisol – Q_10_ supplemented drinking water for 8 weeks after last injection with PQ and dissected 8 weeks after last injection (PQ3 + Ubisol – Q_10_ 8 weeks group). The area of SN which is to be counted is selected in every 6^th^ section of the midbrain (sectioned at 30 microns thickness). The total number of TH positive neurons in the SNpc region was counted at high magnification using the stereology software purchased from the Stereology Resource Centre, Inc., Florida. **(C)** The percentage decrease in TH positive neurons between the saline injected control and PQ injected treated and untreated groups. There is a significant 47% decrease in the TH positive neurons in the PQ3 group in comparison with the saline injected control group (*p < 0.05) and loss of 28% neurons in the PQ3 + Ubisol-Q_10_ 4 weeks group. A 14% decrease in TH positive neurons is seen in the treated PQ3 + Ubisol-Q_10_ 8 weeks (**p < 0.05) indicating significant neuroprotection when compared to the PQ3 group. The bars in the upper (low magnification) panel is 200 μm and in the lower (high magnification panel) 20 μm.

The brain CoQ_10_ levels were measured in rats which were given a 1 h access to Ubisol-Q_10_ supplemented water (at a concentration of 50 μg /ml) after a 24 h period of water deprivation. During this time rats drank on average 10 ml of solution containing 500 μg of CoQ_10_. Animals were sacrificed at different time points after the Ubisol-Q_10_ intake, CoQ_10_ was extracted and analysed by HPLC. The results are shown in Figure 1. We observed a modest, but time dependent elevation of CoQ_10_ in the brain, peaking at 3 h post-feeding, with values 30-50 % higher than the basal level, suggesting that its transfer to the brain parenchyma and subsequent metabolic turnover was taking place. A question whether these changes were sufficient to achieve a therapeutic neuroprotection against PQ was examined below (Figures 2, 3 and 4).

### Paraquat model of progressive neurodegeneration

Long Evans Hooded rats were used to develop a model of progressive neurodegeneration. The rats were given 5 intraperitoneal injections of PQ (10 mg/ kg b.w./injection), one injection every five days over a period of 20 days. Animals were sacrificed at different time points for up to 8 weeks post-PQ exposure. Midbrain sections were prepared, immunostained with anti-tyrosine hydroxylase antibody and TH- positive neurons were counted using a stereologer, in an unbiased manner. As shown in Figure 2, a substantial percentage of DA neurons, i.e., close to 18%, were lost during the PQ injection period (PQ1 group). The neurons continued to die over the next several weeks reducing the number of TH-positive neurons by 43% at the end of week 4 (PQ2 group) and 47% by end of week 8 (PQ3 group) post-PQ exposure. The results confirmed that PQ triggered progressive neurodegeneration in this strain of rats, mimicking to some extent, changes occurring in Parkinsonian patients. This model is probably closest to what happens in humans as one month in a rat’s lifetime is equivalent to 2.5 years in human [20]. Therefore, this model was used to assess a therapeutic neuroprotection of CoQ_10_.

### Therapeutic intervention with Ubisol-Q_10_

We have previously shown that prophylactic treatment with Ubisol – Q_10_ effectively protects rat brain from PQ toxicity [18]. In this study we applied the Ubisol-Q_10_ intervention after the completion of PQ injections. By this time, neurodegenerative processes in the brain had already triggered (Figure 2). The PQ-treated group of rats was placed on Ubisol-Q_10_ supplemented drinking water (containing 50 μg/ml of CoQ_10_) for 4 weeks (PQ2 + 4 wks Ubisol-Q_10_ group). This treatment began when nearly 18% of SN neurons were already lost (PQ1 group, Figure 2), but the question was whether the remaining vulnerable neurons could be saved. The generated data is summarized in Figure 3. The midbrain sections were immunostained with anti- TH antibodies, and the stained neurons were counted using a stereologer in an unbiased manner. As shown above (Figure 2), the PQ- treated rats drinking regular water (PQ2 group) lost over 40% of DA neurons over the period of 4 weeks, whereas rats drinking Ubisol – Q_10_ lost less than 20% (PQ2 + 4 wks Ubisol-Q_10_ group). Clearly, this Ubisol-Q_10_ treatment saved close to 17% of neurons which would have otherwise died as the consequence of PQ exposure. This unprecedented neuroprotection has never been reported in animal models of neurotoxicity and could offer hope to PD patients for better disease management.

We then examined how long Ubisol – Q_10_ supplementation would be required to maintain neuroprotection. In this set of experiments the PQ treated rats were either kept on Ubisol – Q_10_ for the full 8 weeks post-PQ or the treatment was withheld after 4 weeks and rats were given regular tap water for the additional 4 weeks (8 weeks total). There was a significant loss of DA neurons, approximately 47% in comparison with the saline injected control group indicating progressive neurodegeneration over a period of eight weeks (Figure 4). There was also significant neuroprotection in the rats that received the Ubisol – Q_10_ supplemented drinking water for eight weeks post injections. Since the Ubisol – Q_10_ intervention began after nearly 15% of DA neurons were already killed (Figure 2) no further loss of neurons was observed as a result of this intervention (Figure 4, only 14% of neuronal loss recorded). However, if the treatment was withheld after 4 weeks, the neurodegeneration resumed as evidenced by the reduced number of surviving neurons in this experimental group comparing to the group receiving Ubisol – Q_10_ for 8 weeks (28% versus 14%, respectively). Therefore, continuous Ubisol – Q_10_ supplementation was required to maintain the achieved level of neuroprotection.

### Behaviour results

**Figure 5:**
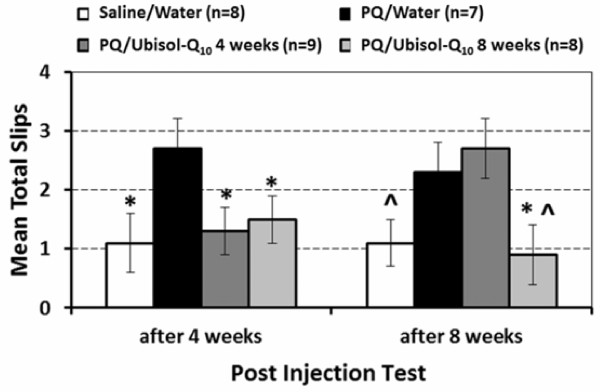
**Mean total number of hind leg slips over three sessions in each post-injection test for each Injection/Treatment group.** Vertical error lines represent ± SEM. Significant difference (*p* < 0.05) between the control or either of the two PQ/ Ubisol – Q_10_ groups and the PQ3 group in each injection test phase are designated by * and between the control and the PQ/Ubisol-Q_10_ 8 weeks group and the PQ3 in the second injection phase by ^.

Deficiency in the motor function is a hallmark of PD. Next, we asked whether loss of DA neurons correlated with the deficiency of behavioural motor function following PQ treatment, and whether motor deterioration was blocked by Ubisol-Q_10_ treatment. We applied a horizontal beam walking test as described in the Method section. Results shown in Figure 5 indicated that the PQ3 group made more leg slips than either the control or the PQ3 + Ubisol-Q_10_ 8 weeks in both the test phases or than the PQ3+ Ubisol-Q10 4 weeks group in the first test phase. The PQ3 + Ubisol-Q_10_ 4 weeks increased its leg slips to the elevated levels of the PQ3 group in the second test phase. Multiple comparisons between groups confirmed that the number of leg slips of the PQ3 group was significantly greater than those of the other three groups (*p* < 0.05) in the 1^st^ test phase but only remained significantly greater than that of the PQ3+/Ubisol-Q_10_ 8 weeks group in 2nd test phase (*p* = .036). Thus even though the observed groups by phase interact was not significant, multiple comparison between groups reveal that only those rats that received Ubisol –Q_10_ in their drinking water over the complete post injection period maintained their superior performance similar to that of rats that were not exposed to potential neurodegenerative effects of PQ. From a behavioral aspect, treatment with the neuroprotectant agent only half way through the post-injection period was not sufficient to maintain its effect to the end of the experiment.

### Toxicity assessment

The data presented above (Figure 4) clearly indicated that long-term treatments with Ubisol – Q_10_ would be required to maintain the neuroprotection. To ensure safety of such long treatments, we carried out a pilot toxicity study, in which rats were maintained on drinking water supplemented with Ubisol – Q_10_ at a dose 10 times higher (60 mg/kg/day) than that used for neuroprotection (6 mg/kg/day) for 2.5 months. During this time, the Ubisol – Q_10_ treated rats never displayed any signs of discomfort, no change in eating, drinking, grooming habits and no difference in body weight in comparison with rats drinking regular tap water over the same time period. At the conclusion of the experiments several tissues were collected and sent for histopathological examination by a board certified pathologist at the University of Guelph. No overt lesions of toxicological significance were observed in the Ubisol-Q_10_-treated animals (data not shown).

## Discussion

For the first time ever, we have effectively established an animal model, using PQ, which accurately demonstrates the chronic and progressive neurodegeneration similar to that in PD patients. Compared to an acute model, our model effectively replicates PD by having slow, chronic degeneration of DA neurons in SNpc. However, progressive neurodegeneration has also been observed with continuous infusion of MPTP using an osmotic pump [21]. The progressive loss was evident in our results over the observed time span, with a decreased number of neurons remaining at each subsequent time interval. Our model allows us to intervene with treatment at any point after the initiation of the disease. Due to this we were also able to demonstrate for the first time, that when Ubisol-Q_10_ is administered therapeutically it effectively halts the progression of the neurodegeneration, even at low dosages. After demonstrating the success Ubisol-Q_10_ has in protecting the neurons therapeutically, it was shown that this treatment needs to be continuous. Halting the administration of the treatment would result in a continuation of the neurodegeneration initiated by the PQ toxin during the injection regime. With bioavailability data, we were also able to show that Ubisol-Q_10_ is effective at increasing the CoQ_10_ levels in the brain. All of the results were supported using behavioural, histochemical, and biochemical methodology.

An animal model of PD was developed when the neurotoxin MPTP was found to cause PD like symptoms and loss of DA neurons by blocking complex I of the electron transport chain [7]. MPTP establishes an acute model of PD, which is not realistic to the natural progression of the disease. Following this, it was found that environmental toxins, rotenone and PQ, can also block complex I of the electron transport chain. Further supported by epidemiological studies, a link between the use of these pesticides and the incidence of PD was established [22]. The development of the PQ animal model of PD is more relevant than the acute MPTP model as it more effectively mimics PD in patients.

In previous research, a variety of dosages and injection regimes of PQ have been used in rat models of PD [14,23]. The downfall of these PQ models was that they were not tested to ensure slow, progressive loss. However, progressive, continuous, and slow neuronal loss in the SNpc was tested and seen in our model during and after our 5 interpretational injection (1 every 5 days for 5 injections). Establishing this model is essential before testing any therapeutic treatment interventions as this is what characterizes PD in patients.

Additional research shows that the pathogenic mechanisms of PD are associated with mitochondrial dysfunction, oxidative stress and altered protein handling [4]. The involvement of mitochondria is considered a key to cell death observed in PD in both sporadic and familial cases.

Previous experiments in our lab have shown that Ubisol-Q_10_ is effective in protecting neurons against toxic insult *in vivo* and can protect DA neurons if administered prophylactically, that is, even before exposure to the environmental toxin, PQ [14]. However, PD is often not diagnosed until symptoms arise, which occurs when almost 50 – 60% neurons are lost.

Once the process is initiated by toxic insult, it is crucial to see if treatment administered therapeutically can halt further neurodegeneration. Ubisol-Q_10_ was tested therapeutically and it showed to have significant protection of the remaining DA neurons after both 4 weeks, and 8 weeks of treatment. This is one of the first experiments to show this. There are multiple explanations which could explain how Ubisol-Q_10_ protects the remaining neurons; initially it is plausible that the combined anti-oxidant nature of the two components of Ubisol-Q_10_ (CoQ_10_ and Vitamin E) could quench the levels of oxidative stress associated with the disease. It was shown that the carrier solution containing vitamin E alone did not have a significant effect on neuroprotection ([14] and data not shown). In other research, it’s been shown that lipid soluble CoQ_10_ (in high dosages) is an effective neuroprotective agent [19]. Past research on Ubisol-Q_10_ has shown it to be effective in stabilizing the mitochondria through inhibiting Bax [24]. Another hypothesis is that it could be protecting the mitochondria by increasing its overall energy output; as CoQ_10_ is naturally found in the electron transport chain.

Previous research using oil soluble CoQ_10_ as a treatment for PD made it into clinical trials in 2011, though failed in phase 2. In their pre-clinical work the oil-soluble CoQ_10_ treatment was tested prophylactically on MPTP induced mouse model [19,25]. The oil soluble CoQ_10_ was shown to be effective, but only at high dosages. A possible explanation to the discontinuation of their clinical trial was because very large dosages (1,600 mg/kg/day) were required to show any neuroprotection. When this dosage is converted to a human dose (averaging 70 kg) they are required to take 112 g/day in order to obtain results, which is beyond the acceptable FDA approved dose for clinical trials (2.4 g). Therefore, in the clinical trial they were not receiving anywhere near the dose required to show positive results. However, our preclinical work, on our more accurate chronological model, treating both prophylactically and therapeutically has shown comparable neuroprotection but at a significantly lower dose (6 mg/kg/day). Therefore, if our dosage was converted for human treatment it would only be 0.42 g/day, which is not only lower then FDA approved amount for clinical trial (2.4 g) but also the approved maximum daily dosage for general supplement intake (1.2 g). Both the oil soluble CoQ_10_ and Ubisol-Q_10_ showed comparable bioavailability when administered, but in order to have comparable quantities in the brain the oil formulation needed to be given in a significantly higher dose [26].

The question remains why Ubisol-Q_10_ is more effective at lower doses than CoQ_10_. It is assumed that the water soluble composition makes absorption into the blood stream easier, therefore, making it possible to cross the blood brain barrier. It is evident in our bioavailability experiment that this formulation does shuttle CoQ_10_ into the brain, due to the increase of 35% after 3 hours. Though, the other significant finding was that once it is in the brain it does not accumulate. This means that there is no build-up of CoQ_10_ in the brain, which could be toxic to the neurons. The natural removal seen explains why when the treatment is withdrawn the effects are no longer sustained. Henceforth, in order to sustain neuroprotection the treatment must be continuous and in doing so neurotoxicity will not result. It is also important to note that the animals in this experiment were allowed to drink Ubisol-Q_10_ supplemented drinking water ad libitum and were not gavaged.

Our study has shown that the withdrawal of Ubisol-Q_10_ leads to continued neurodegeneration, which was triggered by the toxin during the injection period. Therefore, Ubisol-Q_10_ does not halt neurodegeneration by acting on the toxin, but rather by supporting the remaining neurons. This experiment was only conducted with sustained treatment over 8 weeks (with 1 month of treatment and a consecutive month of withdrawal). To ensure the results, more research needs to be conducted over longer time spans. Though the current data found supports sustained treatment regiments in order to withstand neurodegeneration. These findings were also supported by the behaviour data which shows that animals provided with Ubisol-Q_10_ treatment for a longer duration perform better throughout in the beam test compared to the animals where the treatment was withdrawn.

## Conclusion

In conclusion we have shown that the PQ rat model of PD we used in the study shows slow progressive loss of DA neurons and hence mimics what is seen in patients suffering from PD. Our formulation can prevent the death of the remaining neurons in our PD model when administered after the process of neurodegeneration has been triggered and hence could be an effective therapeutic at any stage of the disease. Also, we found that Ubisol-Q_10_ has to be given continuously and cannot be withdrawn in order to continue neuroprotection. Bioavailability studies have shown that even though this formulation is provided at a low dose in order to provide significant neuroprotection, CoQ_10_ does cross the blood brain barrier and there is an increase in the levels of CoQ_10_ in the brain following administration of Ubisol-Q_10_. This formulation of CoQ_10_ is FDA-GRAS approved and preliminary toxicity results show that there is no overt toxicity even when the dose is increased to 10 times the required dose. Ubisol-Q_10_ is an effective neuroprotective agent that could be used effectively to halt the progression of Parkinson’s disease at low doses.

## Methods

### Animal care

All procedures involving animals were carried out in accordance with the Canadian Council for Animal Care guidelines and approved by the University of Windsor’s Animal Care Committee. Three months old male Long Evans Hooded rats were purchased from Charles River Laboratories. Rats in the same treatment group were housed together (3–4 per cage) for convenience and in order to prevent any hierarchy that could arise due to the extent of neurodegeneration. The rooms that housed the rats were maintained at 20°C in a reversed 12 h:12 h dark light cycle.

### Paraquat neurotoxicity model and Ubisol-Q_10_ treatments

Rats received 5 intraperitoneal injections of PQ at a dose of 10 mg/kg body weight/injection dissolved in phosphate buffered saline (PBS), one injection every 5 days over a period of 20 days. Control rats received intraperitoneal injections of PBS alone. Brain tissue was examined immediately after the last PQ injection and, subsequently, 4 weeks and 8 weeks later. Supplementation of drinking water with Ubisol-Q_10_ at a concentration of 200 μg/ml (equivalent to 50 μg CoQ_10_/ml) begun on the day of the last PQ injection and it was continued for either 4 weeks or 8 weeks. Fresh drinking solutions were provided every second day. Sterile stock solution of Ubisol-Q_10_ at 200 mg/ml (equivalent to 50 mg CoQ_10_/ml) was provided by Zymes LLC (Hasbrouck, NJ). At the conclusion of experimental treatments, rats were perfused with Tyrodes buffer containing heparin, the tissues were fixed with 10% formalin, and the brains extracted and stored in the 10% formalin until processing for immunohistochemistry.

### CoQ_10_ bioavailability study

Rats were deprived of water for a period of 24 hours prior to a full 1 h access to drinking water supplemented with Ubisol-Q_10_ at a concentration equivalent to 50 μg CoQ_10_/ml. The rats were sacrificed at 1, 3 and 6 h after the feeding. Brain tissue was collected and CoQ_10_ content was measured as previously described [27-29]. Briefly, samples were homogenized in cold PBS and subjected to repeated freezing/thawing steps to disrupt protein/lipid complexes. CoQ_10_ was extracted and analysed by HPLC following separation on a TSK-GEL ODS-100S column (4.6 mm × 150 mm, 7 μ particle size, TOSOH Biosep LLC, Montgomeryville), equipped with a 1 mm C18 guard column (Optimize Technologies Inc., Oregon City, OR). Absorbance at 275 nm was monitored and recorded using Beckman System Gold Software.

### Toxicity study

A group of rats (4 rats) were kept for 2.5 months on drinking water supplemented Ubisol- Q_10_ at a concentration 2 μg/ml (equivalent to 50 μg/ml of CoQ_10_) or 10 times the dose used in the neuroprotection study. Animals were weighed once a week to ensure their health. The rats were then perfused with heparin containing Tyrodes buffer and formalin fixed tissue – heart, lung, liver and kidney were sent to the Animal Health Laboratory, University of Guelph. Hematoxylin & Eosin -stained histological sections of the tissues were evaluated by a board-certified veterinary pathologist.

### Rat horizontal beam walking test

All rats were assessed for performance on a horizontal beam-walking test for motor skills/motor deficits as measured by leg slips. The aluminium beam was 1.68 metres in length, 2 centimetres in width and 0.75 metres from the ground. A mirror was placed behind the beam, measuring 1.78 metres in length and 0.3 metres in height. Four weeks after the last injection, rats underwent one trial per day for four consecutive days (one training trial and three test trials). Eight weeks after the last injection another three test trials were performed (one trial per day). In the training trial, rats ran down the beam to the holding cage on a flat platform three times, each time with different distances between the holding cage and starting position. The first position was a quarter of the beam length, the second was half, and the last was the entire distance of the beam. This last distance is where mice were placed for the subsequent test trails.

Rats received a small slice of apple in the holding cage located on a table at the end of the beam. The rat had up to 2 minutes to cross the beam. The test trials were recorded using a standard video camera, located 2 metres perpendicular to the beam. The number of hind leg slips made from either leg during each test trial was later noted from viewing the recorded video clips. The number of limb slips for each rat was summed over the three test sessions in each phase because rats made too few slips in each session to analyse this behaviour over trials within each session. The statistical analysis of each rat’s total number of leg slips over each test series was carried by a two-way ANOVA (Groups x Test phase with repeated measures on the second factor). Effects from these analyses were considered significant at *p <* 0.05. Post-hoc comparisons between groups at each test phase were carried out by Least Squares Difference post-hoc multiple comparisons test multiple comparisons and significant differences between groups were considered at *p* < 0.05 (one-tail) based on the prediction that rats not given post-injection Ubisol-Q_10_ in their drinking water would show deficits associated with PQ-induced neurodegeneration.

### Immunohistochemistry

The brains kept in 10% formalin were transferred to 30% sucrose (w/v) three days before sectioning. The midbrain region was sectioned at a thickness of 30 μm and 64 sections were collected in total. The sections were subjected to immunohistochemistry with anti-tyrosine hydroxylase antibody (1:1000 dilution) purchased from Pel-Freeze Biologicals, USA. Prior to overnight incubation with the primary antibody at 4°C, the sections were incubated in 1% H_2_O_2_ for 5 minutes to block endogenous peroxidase, DAKO universal blocking solution (purchased from Diagnostics Canada Inc., Mississauga) for 30 minutes and in normal goat serum (prepared as per instructions on anti-rabbit Vecstatin ABC Kit, Vector Laboratories) for 30 minutes in order to block the binding of non-specific goat IgG. The sections were washed in Tris buffered saline (TBS) twice for 5 minutes between the blocking steps in order to remove any excess blocking reagents. After overnight incubation the slides were washed in TBS twice and incubated in biotinylated anti-rabbit IgG raised in goat (anti-rabbit Vecstatin ABC Kit) for 1.5 hours. The slides were again washed twice for 5 minutes with TBS, following which they were incubated in avidin biotin complex (ABC reagent) for 45 minutes. Then the two five minute washes with TBS were repeated, and the peroxidase substrate 3, 3′ diaminobenzidine (DAB) prepared as per the instructions to specifically stain the DA neurons. The sections were then dehydrated with 95% ethanol and xylene and coverslipped to be able to visualise under the microscope.

The number of TH-positive neurons in the SN were counted using the Stereology Software provided by the Stereology Resource Center, Chester, MD as previously described [30,31]. The SN region was outlined at low magnification and the neurons were counted at high magnification.

### Statistical analysis

Differences among means were analysed using one way analysis of variance (ANOVA) and pairwise comparisons between means were analyzed by *post hoc* Bonferroni’s multiple comparison test. The software used was GraphPad Prism ver. 4.0.

## Abbreviations

PD: Parkinson’s disease; SN: Substantia nigra; SNpc: Substantia nigra pars compacta; DA: Dopaminergic; PQ: Paraquat; MPTP: 1-methyl-4-phenyl-1,2,3,6-tetrahydropyridine; MPP+: 1-methyl-4-phenylpyridinium; NRC: National Research Council; PEG: Polyethylene glycol; PBS: Phosphate Buffered Saline; TBS: Tris Buffered Saline; GLP: Good lab practices; ABC: Avidin Biotin Complex; ANOVA: Analysis of variance; FDA: Food and Drug Administration; GRAS: Generally recognised as safe.

## Competing interests

Dr Shelley Weinstock is paid employee of the Zymes LLC, the company which has the licence for Ubisol - Q_10_, hence the financial interest.

## Authors’ contributions

KM, MS, JKS, JC and SP contributed to the planning and execution of the experiments and writing the manuscript. KM, SL, KH, HM and PL were involved in performing injection and feeding of different regiments, dissections, immunohistochemical analysis and biochemical analysis. JC, CK and DL were involved in the design and execution of behavioural tests, analysis of rotorod results and animal care. MS, HBB and SW prepared water-soluble CoQ_10_ and placebo formulations. All authors read and approved the final manuscript.
