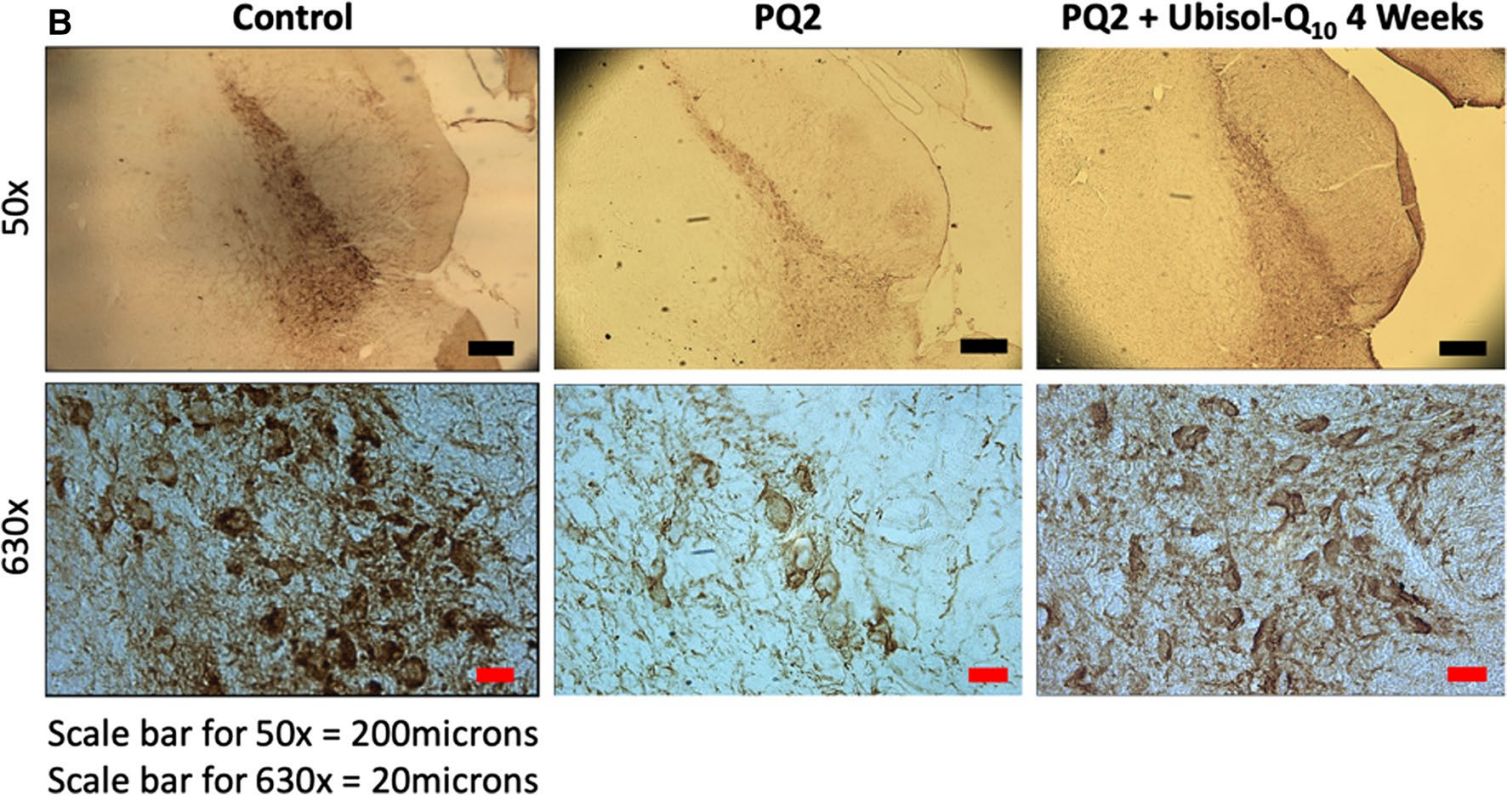# Correction to: Orally delivered water soluble coenzyme Q10 (Ubisol-Q10) blocks on-going neurodegeneration in rats exposed to paraquat: potential for therapeutic application in Parkinson’s disease

**DOI:** 10.1186/s12868-021-00684-7

**Published:** 2021-12-20

**Authors:** Krithika Muthukumaran, Samantha Leahy, Kate Harrison, Marianna Sikorska, Jagdeep K. Sandhu, Jerome Cohen, Corrine Keshan, Daniel Lopatin, Harvey Miller, Henryk Borowy-Borowski, Patricia Lanthier, Shelly Weinstock, Siyaram Pandey

**Affiliations:** 1 Chemistry and Biochemistry, University of Windsor, 401 Sunset Ave, Windsor, ON Canada; 2 Psychology, University of Windsor, Windsor, ON Canada; 3 Translational Bioscience, Human Health Therapeutics Portfolio, National Research Council Canada, Ottawa, ON K1A 0R6 Canada; 4 Zymes LLC, Hasbrouck Heights, NJ USA

## Correction to: BMC Neuroscience (2014) 15:21 https://doi.org/10.1186/1471-2202-15-21

Following publication of the original article [1], it was reported that there was an error in Figs. 2B and 3B. One of the figure panel (PQ2 High Magnification) from Fig. 2B was mistakenly duplicated in Fig. 3B (PQ2 High Magnification) Also a high magnification panel of Fig. 2B (control) was put in in Fig. 3B (PQ + Ubisol Q_10_ 4 weeks). We have corrected this error.

The corrected versions of Figs. 2B and 3B are included in this erratum.

Figure 2B:
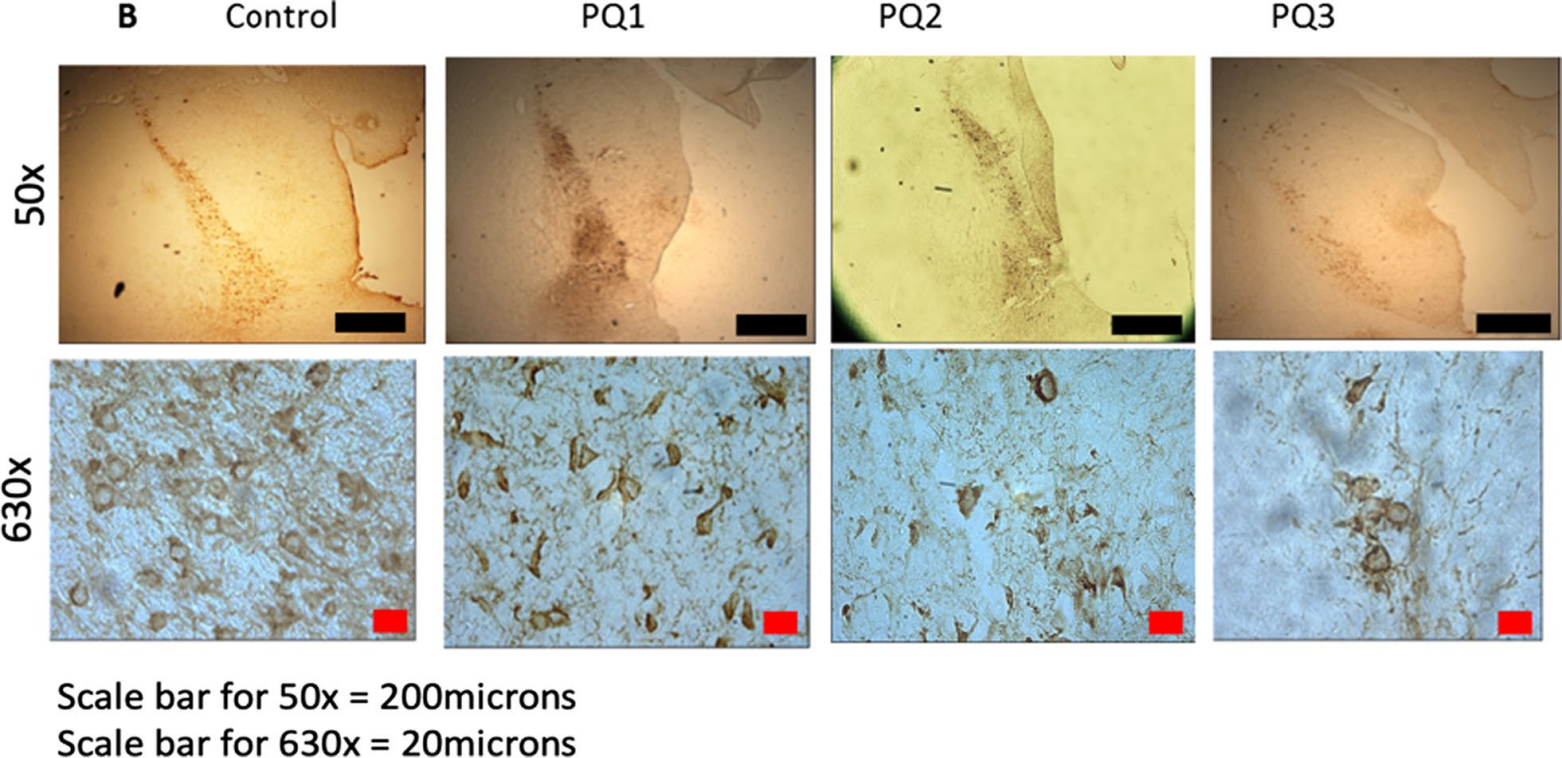


Figure 3B: